# Primary HSV-2 Infection Complicated by Radiculomyelitis in a Young Immunocompetent Female Patient with Inherited Chromosomally Integrated HHV-6: A Case Report

**DOI:** 10.3390/v14091979

**Published:** 2022-09-07

**Authors:** Marie Thérèse Ngo Nsoga, Alice Accorroni, Aline Mamin, Manuel Schibler

**Affiliations:** 1 Division of Infectious Diseases, Department of Medicine, Geneva University Hospitals, 1205 Geneva, Switzerland; 2 Faculty of Medicine, University of Geneva, Rue Michel Servet 1, 1206 Geneva, Switzerland; 3 Division of Neurology, Department of Clinical Neurosciences, Geneva University Hospitals, 1205 Geneva, Switzerland; 4 Laboratory of Virology, Division of Laboratory Medicine, Diagnostic Department, Geneva University Hospitals, 1205 Geneva, Switzerland

**Keywords:** Herpes simplex virus 2, radiculomyelitis, Elsberg syndrome, inherited chromosomally integrated HHV-6

## Abstract

Background: HSV-1, HSV-2 and VZV are alpha Herpesviruses, neurotropic viruses that are associated with various neurologic complications upon primary infection or reactivation. Cases of myelitis and radiculomyelitis are rare and appropriate etiologic diagnoses can be tricky. Case presentation: Here we describe the case of a young immunocompetent woman who developed painful and extended vesicular genital lesions, with subsequent radiculomyelitis. HSV-1/-2 PCRs in the cerebrospinal fluid were misleadingly negative, whereas HHV-6 PCR was positive. Positive anti-HSV-2 IgM and IgG in serum was consistent with HSV-2 primary infection. On the other hand, the detection of HHV-6 DNA was explained by inherited chromosomally integrated HHV-6. The clinical course was favorable with high-dose IV acyclovir and corticosteroids. Conclusion: HSV-2-related radiculomyelitis is a rare clinical entity, which can be difficult to diagnose. In this case report, the causative virus was not detected in the patient’s CSF, whereas HHV-6 DNA, non-pathogenic in this situation, was paradoxically positive. The diagnosis was based on the clinical features typical for HSV-2 primary infection, confirmed by the serology results. The delay between the genital lesions and the appearance of the radiculomyelitis, along with the absence of HSV-2 detection in the CSF, suggests a possible immuno-mediated physiopathological process. As for the HHV-6 DNA detection in the patient’s CSF, it was explained by inherited chromosomally integrated HHV-6. This case illustrates how both negative and positive clinical virology results need careful interpretation according to the clinical findings.

## 1. Introduction

Herpes simplex virus 1 (HSV-1) and 2 (HSV-2) are members of the alphaherpesvirus subfamily within the herpesvirus family. These viruses cause oro-labial and genital primary infections, which are mostly asymptomatic, and subsequently enter a latency phase in sensory neurons. From there, viral reactivations can occur, some of which can be clinically apparent. HSV-2 infections are most often sexually acquired and seroprevalence increases from 20–30% at age 15–29 years to 35–60% at age 60 years [1,2]. HSV-2 is a well-known pathogen of the central nervous system and causes various manifestations including meningitis, which is often recurrent and usually mild, encephalitis, radiculitis and radiculomyelitis [3,4,5,6,7].

According to the literature, HSV-2 represents the second or third most common viral cause of meningitis, after enteroviruses and varicella-zoster virus (VZV) in adult patients [7,8,9,10]. It can infrequently cause encephalitis and myelitis [5,7] and rarely radiculitis and radiculomyelitis, known as the Elsberg Syndrome. In the latter cases, symptoms tend to last from a few days to a few months. Frequently reported symptoms are urinary retention, constipation, erectile dysfunction, anogenital discomfort, paresthesias and anesthesia or flaccid paresis of the lower limbs muscles [11].

Other viruses known to cause radiculomyelitis include HSV-1, VZV, Epstein-Barr virus, cytomegalovirus and enteroviruses. Cerebrospinal fluid (CSF) analysis usually shows a lymphocytic pleocytosis and a slight increase in proteinorachia [11]. The proportion of positive virologic polymerase chain reaction (PCR) findings is quite low [12]. Serology and the intrathecal synthesis of virus-specific antibodies can help for diagnosis. Acyclovir and corticosteroids represent the treatment of choice although there is little evidence of their effectiveness. There is no consensus regarding the duration of treatment that varies significantly among case studies/reports [13,14].

## 2. Case Presentation

A healthy 28-year-old woman presented with vesicular genital lesions. She was diagnosed with primary HSV-2 infection, as confirmed by a positive HSV-2 PCR performed on a genital lesion swab. She was treated with valacyclovir 500 mg twice a day for 10 days. Ten days after the end of treatment, she experienced two episodes of urinary retention episodes, requiring intermittent urinary catheterization, as well as paresthesia involving the soles of the feet, as well as her toes, vulva and anus. 

On admission, she was alert and oriented in time, space and situation (Glasgow score: 15), hemodynamically stable and afebrile. She did not have any signs of meningitis, cranial nerves, deep tendon reflexes, and motor and sensory exams were normal apart from the persistence of paresthesia. The anal tone was preserved. The rest of the examination was unremarkable.

The serum leukocyte count was normal (7.2 G/L; normal value: 4–11 G/L), and the C-reactive protein was <0.30 mg/L (Normal value: 0–10 mg/L). The analysis of the CSF showed a clear fluid, a moderately elevated white blood cell count (58 M/L; Normal value: 0–5 M/L) with a lymphocytic predominance (94%), a discrete proteinorachia at 0.76 g/L (Normal value: 0.15–0.045 g/L) and a discrete hypoglycorachia (2.6 mmol/L; normal value: 2.8–4.0 mmol/L).

The multiplex PCR BioFire^®^ FilmArray^®^ Meningitis/Encephalitis (ME) Panel was negative for HSV-2 and positive for human herpesvirus 6 (HHV-6); of note, the assay used does not distinguish HHV-6A from HHV-6B. The panel results for all other tested pathogens were negative. 

An HSV-1/-2 real-time PCR (rPCR) assay in CSF was also negative, and so was the intrathecal anti-HSV-2 IgG synthesis antibody index. However, HSV-2 serology was positive with an IgM index at 29.3 (threshold 10) and IgG at 7.4 (threshold 1), which was suggestive of primary HSV-2 infection; HSV-1 serology IgM was positive with an IgM index at 24.8 (threshold 10) and serology IgG was negative at 0.3 (threshold 1); Epstein-Barr virus serology IgM was weakly positive at 1.50 (threshold 1) probably due to cross-reactivity with HSV-1. 

The presence of HHV-6 DNA in the patient’s CSF was confirmed by a qualitative rPCR and quantified at 50,000 copies/mL using a quantitative rPCR (qPCR) assay. HHV-6 DNA was also detected and quantified in plasma (26,000 copies/mL), as well as in the patient’s nails, indicating inherited chromosomally integrated HHV-6 (iciHHV-6).

**Figure 1:**
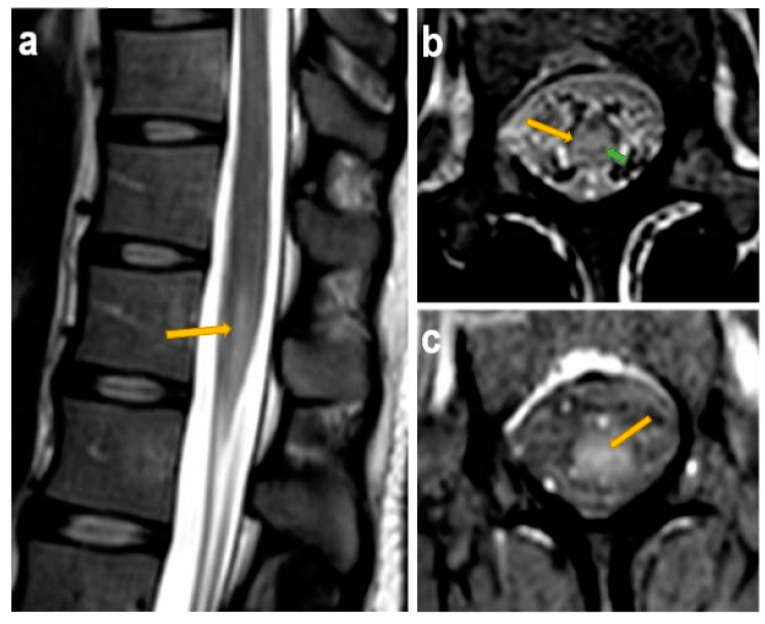
Magnetic resonance imaging of the lumbosacral spine. Sagittal (**a**) and axial (**b**) T2 weighted showing enhancement (yellow arrows), and axial T1-weighted sequence (**c**), showing contrast enhancement of the medullary cone. Panel (**b**) also displays sparing of some nerve bundles (green arrow).

Magnetic resonance imaging of the lumbosacral spine showed evidence of medullary cone myelitis, with possible inflammation at the level of the cauda equina, especially at sacral roots 1 and 2 and thoracic roots 11 and 12 (Figure 1). 

The patient’s final diagnosis thus was primary HSV-2 infection-related radiculomyelitis, with fortuitous iciHHV-6 discovery. 

A treatment consisting of intravenous (IV) acyclovir (10 mg/kg/8 h) associated with IV boluses of methylprednisone (1 g/daily for 3 days) was initiated on admission. IV Antiviral treatment was prescribed for a total of 12 days. Corticosteroid therapy was continued with a tapering regimen of oral prednisone, for a total of 17 days. Twelve days after her admission, the patient described an almost complete resolution of her paresthesias, the urinary catheter was removed without post-micturition residue, and good bladder emptying was observed despite the persistence of a slightly pathological flow curve.

## 3. Discussion

The clinical picture (extended genital vesicular lesions), the positive HSV-2 PCR, the positive anti-HSV-2 IgM and IgG, the latter at a relatively low level in a serum sample collected two weeks after onset of symptoms, indicate a symptomatic primary HSV-2 infection, rather than a reactivation. The latter cannot be completely excluded but seems to be a far less likely hypothesis, essentially given the extension of the genital lesions. Furthermore, in the case of an HSV-2 reactivation, the anti-HSV-2 IgM titer would have been expected to be either negative or only slightly positive, while the anti-HSV-2 IgG titer would have been expected to be higher. The anti-HSV-1 (and anti-EBV) IgM positivity is most likely explained by cross-reactivity with anti-HSV-2 IgM, which is a well-described phenomenon regarding herpesviruses serology in general [15]. 

In this case, the specific HSV-2 PCR on genital lesions confirmed that the causative virus was HSV-2 and not HSV-1. The occurrence of radiculitis 10 days after the onset of the genital HSV-2-related symptoms is very suggestive of the so-called Elsberg syndrome, which is typically attributed to HSV-2. In this entity, HSV-2 DNA is not always detected in the CSF, as described by Savoldi and al [12]. The absence of HSV-2 DNA detection in some of Elsberg syndrome CSF samples might be explained by an immuno-mediated pathophysiological process underlying radiculomyelitis, although there was no measurable intrathecal anti-HSV-2 IgG production, rather than direct viral damage.

The case of this young patient presenting with primary HSV-2 infection-associated radiculomyelitis is educational for several reasons. First, this presentation is unusual, and it underlines the importance of medical history and clinical examination in making the correct diagnosis. Second, this case reminds clinicians that careful interpretation of microbiological and laboratory results, in general, is critical to avoid diagnostic pitfalls, especially when such results are misleading, as it is the case in the present report. Indeed, the putative causative agent, namely HSV-2, was not detected in the patient’s CSF, whereas HHV-6 DNA was detected by several PCR assays. HHV-6 is a known neuropathogen, even in adults, but is typically only pathogenic in highly immunosuppressed patients, and usually causes limbic encephalitis, rather than radiculomyelitis [16]. Therefore, iciHHV-6, leading to the presence of HHV-6 DNA in all nucleated cells and thus in virtually all clinical samples, should be suspected whenever a positive HHV-6 PCR result does not explain a patient’s clinical presentation. In this case, this entity was confirmed by HHV-6 DNA detection in the nails. Other laboratory techniques used to confirm iciHHV-6 are discussed elsewhere [17]. 

Regarding HSV-2-related radiculomyelitis treatment, it is important to keep in mind that it is not well studied due to the rarity of this illness. The antiviral-corticosteroid combination is rather based on the idea that both direct viral and inflammatory mechanisms could be implicated in the pathogenesis. However, despite treatment, the clinical outcome is not always favorable, and recovery is sometimes only partial [12].

## 4. Methods

PCRs on CSF were performed using the Biofire^®^ FilmArray^®^ Torch system with the Meningitis/Encephalitis (ME) Panel (Biomérieux, Petit-Lancy, Switzerland). This microfluidics-based multiplex assay allows for simultaneous testing for 14 neuropathogens, among which 7 viruses, 6 bacteria and 1 yeast. For HHV-6 quantification, DNA from CSF or plasma is extracted using the eMag system (Biomérieux, Petit-Lancy, Switzerland) and PCR is performed using the HHV6 R-Gene^®^ kit (ref 69-006B, Biomérieux France, Craponne, France). Standards of known concentrations are analyzed in parallel to generate a calibration curve which allows for the quantification of HHV6 DNA in copies/mL. Regarding DNA extraction from the nails, nail fragments are digested overnight with Proteinase K and DTT buffer at 56 °C. The next day, the digest is filtered with the Lyse&Spin Basket kit (ref 19597, Qiagen, Hombrechtikon, Switzerland) and DNA is extracted with the QIAamp DNA Mini Kit (ref 51304, Qiagen, Hombrechtikon, Switzerland). An in-house qualitative PCR is used for HHV-6 DNA detection (design and PCR conditions on request).

HSV-1 and HSV-2 serology was performed using the following ELISA assays: HSV-1 IgG and HSV-2 IgG kits from GenBio (SanDiego, CA, USA), and HSV-1 IgM and HSV-2 IgM kits from Novagnost (Novatec, Dietzenbach, Germany). These assays were all run on the Dynex Agility System. The cut-off was set at 1 for IgG and 10 for IgM by the supplier.

## Data Availability

Not applicable.

## Abbreviations

HSV-1	Herpes simplex virus 1
HSV-2	Herpes simplex virus 2
VZV	Varicella-zoster virus
CSF	Cerebrospinal fluid
PCR	Polymerase chain reaction
HHV-6	Human herpesvirus 6
iciHHV-6	Inherited chromosomally integrated HHV-6
rPCR	Real-time polymerase chain reaction
qPCR	Quantitative polymerase chain reaction
IV	Intravenous
